# Tropical Livestock Units: Re-evaluating a Methodology

**DOI:** 10.3389/fvets.2020.556788

**Published:** 2020-11-20

**Authors:** Peregrine Rothman-Ostrow, William Gilbert, Jonathan Rushton

**Affiliations:** Institute of Infection, Veterinary and Ecological Sciences, University of Liverpool, Neston, United Kingdom

**Keywords:** tropical livestock unit, TLU, biomass, livestock, food security

## Abstract

The dynamic between humans, livestock, and wildlife is evolving owing to growth in populations, a finite global landmass, and shifting climatic conditions. This change comes with certain benefits in terms of food security, nutrition, and livelihoods as livestock populations increase, but is not without risk. The role of livestock in infectious disease emergence, environmental degradation, and the development of antimicrobial resistance is becoming more apparent. An understanding of these risks and development of mitigation tactics, especially in low- and middle-income countries where the pace of change is most rapid, is increasingly based on comprehensive models and tools built to map livestock populations at the global, regional or national level. Translation of model estimates into evidence is often underpinned by a quantification of livestock biomass to support policy development and implementation. This paper discusses the application of the Tropical Livestock Unit in the context of measuring biomass. It examines the established method of calculation, designating all cattle a standard weight of 175 kg, and compares it to two proposed alternatives. In doing so, the potential to refine estimates of biomass in low and middle-income countries is explored, though this concept could be extrapolated to higher income economies as well. Publicly available data from six countries in sub-Saharan Africa was utilized to demonstrate how breed liveweight, herd structures, and growth rates have the potential to dramatically alter the estimates of cattle biomass in each country. Establishing standardized data collection procedures to capture this information on a regular basis would grant a better understanding of the true nature of livestock populations, aid in the development of superior disease prevention and response measures, bolster food security initiatives through improving livestock production, and inform the intelligent management of shared ecosystems to improve conservation and biodiversity.

## Introduction

Driven by growing prosperity and expansion of the world's population, expected to reach 9.7 billion by 2050, demand for animal-derived products is expected to rise considerably over the next century (1, 2). Concurrently, infectious disease emergence, climate change, and loss of biodiversity increasingly threaten food security, human health, and the global economy (3). With Africa expected to contribute 50% to global human population growth, the pace of change, and the escalation of these risks, are expected to be most rapid in low and middle-income countries (LMICs) (4, 5).

Livestock populations in LMICs (as defined by the World Bank) are already seeing a steady increase in numbers alongside population growth and wealth increase (6, 7). In Kenya for example, a country defined as lower-middle-income by the World Bank, cattle populations are projected to increase by 94% and poultry by 375% between 2015 and 2050 (7, 8). Initiatives to expand and intensify production systems as well as improve species production potential are underway in many LMICs (9, 10). Simultaneously, the risks inherent in the rapid transition and concentration of livestock systems are recognized, and attempts to more accurately map and manage populations have been made. Central to mapping efforts, the Gridded Livestock of the World database, initially published in 2007 and now in its third edition, has modeled livestock distribution and density around the world using data compiled through censuses and national statistics, cross-referenced with the Food and Agriculture Organization of the United Nation's FAOSTAT database (11). Researchers have also leveraged this data to inform mapping of global livestock biomass distributions (12–14). The distribution and density mapping efforts exemplify the increasing level of resolution that analytic methods are looking to capture, however that level of granularity is not mirrored in the estimation of biomass by tropical livestock units (TLU), which are foundational in much of the work done in LMICs.

Measuring 250 kg of liveweight, the TLU has been used as the reference point to factor livestock of different species by biomass in LMICs since at least the mid-20th century (15). In his 1982 manual, *Livestock Production Systems and Livestock Development in Tropical Africa*, Jahnke (15) discussed the convenience of being able to quantify a variety of forage-consuming domesticated animal species through the TLU as a means of informing rangeland carrying capacity and stocking rates. The camel, as the largest livestock species in tropical regions at that time, with an average liveweight of 250 kg, was defined as 1 TLU; further conversion factors were established for the remaining species. Cattle were assumed to have an average weight of 175 kg, equating to 0.7 TLU per head, with 0.1 TLU per head allocated for sheep and goats, 0.2 for pigs, 0.8 for horses, 0.7 for mules, 0.5 for asses, and 0.01 for chickens.

The same conversion factors as outlined in the mid-20th century are still in use to quantify the biomass of species today, however, weaknesses in this method of calculating and utilizing the TLU appear abundant. When considering a species, averaging the weight of animals regardless of breed, sex, or age fails to account for vast differences that could be observed when assessing population structures. Doing this precludes any possibility of monitoring change within a species population that may appear as a result of breed or nutritional improvement, or from negative factors such as disease, lack of access to adequate nutrition, or other climatic or environmental variables. In consideration of stocking densities—the original inspiration for developing the TLU—the importance of grasslands as a means of grazing livestock, sustaining wildlife, reducing soil erosion, and mitigating greenhouse gasses has grown increasingly important and it is evident that assessment of impacts must become more precise (16). Yet, all cattle, regardless of age, breed, sex, or agricultural purpose (e.g., meat vs. dairy) are presently still estimated to average 175 kg liveweight; all small ruminants are averaged at 25 kg per head, and all chickens averaged at 2.5 kg per bird. Additionally, this method of calculating biomass does not consider differences in feed conversions, growth rates, or production efficiency specific to different animals. Even in early mentions of the TLU and the animal unit or animal unit equivalent (similar biomass measurement tools used in the United States for informing stocking densities) it was acknowledged that an animal's metabolic weight, fertility rate, and the herd structure must be considered in the context of potential intake to generate the most accurate calculations (15, 17, 18). It would seem then to be completely erroneous, for example, to assume that 70 chickens would have the same value, nutritional needs or greenhouse gas emission potential as one cow.

Increasingly, however, livestock density patterns and biomass estimates using the TLU are being utilized to underpin evidence in research on a variety of factors: to identify at-risk populations in consideration of climate change and impacts on food security; to determine land carrying capacity; to examine stocking rates for the purpose of supporting proposals for livestock development projects; and as an indicator and predictor of wealth or diversification of income (14, 19–24). Livestock biomass has also been explored extensively in relation to greenhouse gas emissions (GHG) either directly from animals or as a result of their excrement or impact on soils (24, 25). Physical pressures on a landscape are mitigated by a variety of factors, however, it is evident that biomass, along with whether the animal is a ruminant or monogastric herbivore are key components, particularly in the production of methane (26, 27).

The availability of data on animal weights, breed characteristics, and population numbers is greater now than in the past through the work of research groups, breed societies, and aggregation by databases such as FAOSTAT and FAO Domestic Animal Diversity Information System (DAD-IS) (11, 28). In response to these factors, this paper explores alternative methods for estimating population biomass as a comparison to the traditional TLU estimation method, demonstrating the potential impact on total biomass estimates. An improved estimation of biomass has vital and far reaching applications in the monitoring of the health, nutrition, and environmental impacts of livestock production in LMICs and will be an important scale factor in the estimation of economic impact of disease through the Global Burden of Animal Diseases (GBADs) program (29).

## Methods

To explore how traditional biomass estimates differ when compared with estimated average cattle liveweight in each country, FAOSTAT and FAO DAD-IS databases for the years 2010–2020 were cross-referenced. Countries that were selected reported all four of the following data points in the same calendar year:

- Population head of cattle (DAD-IS; FAOSTAT);
- Carcass weight data (FAOSTAT);
- Head of cattle per breed (DAD-IS), totaling at least 91% of FAOSTAT population;
- Weight data for males and females of each breed (DAD-IS).

FAOSTAT herd population estimates and DAD-IS population data were reviewed to verify they were within a 90% identical range. Only six sub-Saharan African countries matched the search criteria: Burundi (2013), Malawi (2013), Mali (2015), Mozambique (2018), Niger (2018), and Senegal (2019).

**Figure 1:**
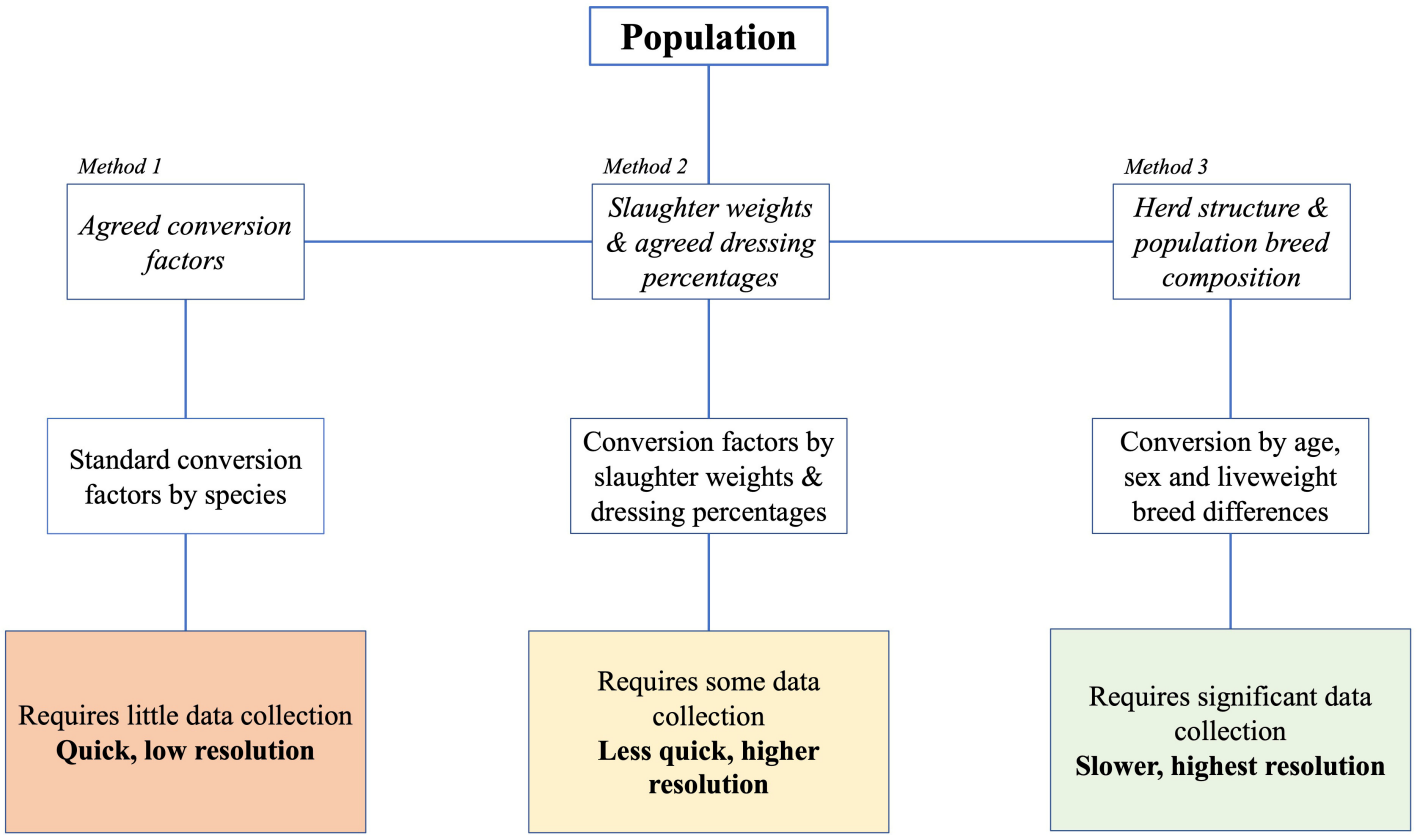
Estimating tropical livestock units of a given [cattle] population requires an understanding of population biomass. Historically, herd biomass has been estimated by multiplying the population number by an average liveweight estimate of 175 kg (Method 1). We argue that while Method 1 may be expedient, it does not accurately represent population biomass. Therefore, we propose that an understanding of herd structure and breed composition, as well as an understanding of the age, sex, and breed liveweight differences is essential to a complete understanding of population biomass and conversion to an informed TLU calculation (Method 3). In the absence of data required to implement Method 3, an interim solution is use of slaughter weights and agreed dressing percentage to inform liveweight biomass estimation (Method 2).

Using this data, three ways of calculating livestock biomass were compared (Figure 1):

1. Use of population estimates (DAD-IS) with standard weight-based TLU conversion values.
2. Use of population estimates with liveweight defined by dressed carcass weight at slaughter (FAOSTAT) and a standard dressing percentage.
3. Use of population estimates by breed and associated weight data (DAD-IS) with an assumed herd structure.

### Method 1

As a baseline for comparison, average cattle liveweight (175 kg) was divided by 250 kg to convert to TLUs by the standard method and then multiplied by population (head) in each country (Equation 1). Cattle population was represented using DAD-IS estimates (>91% identical to FAOSTAT population estimates) to streamline further analysis comparisons between the methods.

(1) $$\begin{array}{l} TLUs=\left(\frac{175}{250}\right)\times population \end{array}$$

### Method 2

Using year-specific average dressed carcass weights obtained from FAOSTAT for each country, carcass weight was divided by standard-use cattle dressing percentage of 55% to find average liveweight (Equation 2). Average liveweight was then divided by 250 for conversion to TLU.

(2) $$\begin{array}{l} Average\;liveweight=\;\frac{Dressed\;carcass\;weight}{Dressing\;percentage} \end{array}$$

### Method 3

The third method used breed population and weight data obtained from DAD-IS for each country. The cattle population of each country was compartmentalized by breed, such that for each breed, *n*_*i*_ represents the population of breed *i* within the country. Within each breed, the population was then further divided into calves, young stock, adult males, and adult females: the proportion of each being *P*_*c*_, *P*_*y*_, *P*_*b*_, and *P*_*f*_, respectively. The average liveweight for each age category (*W*_*c*_, *W*_*y*_, *W*_*b*_, and *W*_*f*_) was then weighted by proportion to calculate an average liveweight for the breed (*W*_*i*_) (Equation 3).

(3) $$\begin{array}{l} W_{i}=\;P_{c}\cdot W_{c}+\;\frac{P_{y}\left(W_{b}+W_{f}\right)}{2}+\;P_{b}\cdot W_{b}+P_{f}\cdot W_{f} \end{array}$$

To illustrate, a population structure of 20% calves, 25% young stock, 35% adult females and 20% adult males was assumed. Calves were defined as birth to 6 months of age and were assigned an average weight of 50 kg; young stock were defined as 7–18 months of age and assumed to average 50% of adult weight with an even sex split. The contribution of each breed to biomass was then calculated as the product of *W*_*i*_ and *n*_*i*_. Each breed biomass was then summed by country to estimate a total biomass in kilograms, which was converted to TLU as 1 TLU = 250 kg.

## Results

A review of the results shows that in all six of the countries, biomass estimates were considerably higher when alternative methods of calculation are applied. Results of the traditional method of calculating herd biomass by a standard average of 175 kg (section Method 1) are shown in Table 1 alongside total estimated biomass for each country.

**Table 1:** Herd biomass estimates derived from a standard average of 175 kg per head.

**Country**	**Year**	**Head**	**Biomass by standard average (head × 175kg)**	**TLUs**
Burundi	2013	690,000	120,750,000	483,000
Malawi	2013	1,241,749	217,306,075	869,224
Mali	2015	9,747,326	1,705,781,963	6,823,128
Mozambique	2018	2,007,936	351,388,800	1,405,555
Niger	2018	13,788,596	2,413,004,300	9,652,017
Senegal	2019	3,642,866	637,501,463	2,550,006

Data obtained from the FAOSTAT database for average dressed carcass weights by country in Method 2 illustrate that carcass weight was greater than Method 1 liveweight estimates (all cattle equal to 175 kg) in Burundi, Niger, and Senegal which reported average dressed carcass weights of 200, 278, and 188 kg, respectively. Conversion of all average carcass weights to liveweights using Method 2 found animals averaged between 224 and 505 kg liveweight (Table 2) which, when compared with Method 1 standard liveweight of 175 kg, yielded between 116 and 289% greater [total] biomass across the countries reviewed. This is illustrated in **Table 4** where biomass per country obtained through Method 1 is shown as a percent of biomass derived through Method 2.

**Table 2:** Total biomass derived through conversion of all average carcass weights to liveweights using a standard dressing percentage of 55%.

**Country**	**Average carcass weight (kg)**	**Liveweight (kg)**	**Converted to TLU**	**Total national biomass (kg)**	**TLUs**
Burundi	200	364	1.5	250,909,091	1,003,636
Malawi	112	204	0.8	252,865,251	1,011,461
Mali	123	224	0.9	2,179,856,430	8,719,426
Mozambique	162	295	1.2	591,428,422	2,365,714
Niger	278	505	2.0	6,969,508,524	27,878,034
Senegal	188	342	1.4	1,245,197,662	4,980,791

Analysis of data obtained from DAD-IS found between 1 and 12 different breeds represented in each country with reported liveweights for adult females ranging from 230 to 800 kg and adult males ranging from 300 to 1100kg. Analysis of Method 3 illustrated that application of herd structure in the context of breed data yielded greater total biomass (Table 3) and larger average liveweights than Method 2 in all but one country (Niger) (Table 4). A comparison of results between Methods 1 and 2 found the standard method of calculation captured between 35 and 86% of total biomass compared to Method 2, and between 41 and 75% of total biomass compared to Method 3 (Table 4).

**Table 3:** Biomass estimates derived through by compartmentalization of specific country breed and associated weight data into an assumed herd structure.

**Country**	**Average Liveweight (kg)**	**Total biomass (kg)**	**Total TLUs**
Burundi	430	296,866,750	1,187,467
Malawi	233	289,793,173	1,159,173
Mali	265	2,587,612,287	10,350,449
Mozambique	455	912,737,392	3,650,950
Niger	282	3,889,560,765	15,558,243
Senegal	365	1,328,395,973	5,313,584

**Table 4:** Comparison of biomass estimation methodologies.

**Country**	**Year**	**Average Animal Liveweight (kg)**			**Ratio of total biomass**	
		**Method**			**Method 1 as a % of Method 2**	**Method 1 as a % of Method 3**
		**1**	**2**	**3**		
Burundi	2013	175	363.6	430.2	48%	41%
Malawi	2013	175	203.6	233.4	86%	75%
Mali	2015	175	223.6	265.5	78%	66%
Mozambique	2018	175	294.5	423.6	59%	38%
Niger	2018	175	505.5	282.1	35%	62%
Senegal	2019	175	341.8	364.7	51%	48%

## Discussion and Conclusions

The intention of this paper was to fulfill two purposes. First, to ascertain whether deviations from an average cattle liveweight of 175 kg would yield significant changes in biomass estimates in sub-Saharan Africa, and second, to use publicly available datasets to generate estimates of average liveweight for comparison to the 175 kg benchmark. The investigation demonstrated that there is capacity to introduce a greater degree of fidelity into biomass estimates for livestock populations. The data extracted from the DAD-IS and FAOSTAT databases are suggestive of a trend toward under-estimation of cattle liveweight in the current biomass estimation methodology, in particular in the central and southern African countries examined here, and in those countries with a greater introduction of exotic genetics. Indeed, failure to update average liveweights when considering TLUs fails to recognize the significant efforts made by various groups to improve the genetics and nutritional input of livestock species in tropical regions, an agenda that is hailed ever more frequently at the policy table.

It is acknowledged that some limitations must be taken into consideration when appraising our analysis. First, Method 2 is based on the assumption that dressed carcass weights are directly representative of average liveweight in the population at large, and that all cattle produce a dressing percentage averaging 55%. These are clearly dubious assumptions to make, given that this sample is likely to include both emergency and regular slaughter animals, as well as recent imports through trade, and unlikely to include many calves. The choice to use an average cattle dressing percentage (also referred to as killing-out percentage) of 55% could further inhibit calculation of exact liveweight estimations. A dressing percentage is calculated as the proportion of animal mass that is considered fit for consumption. While studies have attempted to estimate dressing percentage in different breeds and environments, it is difficult to extrapolate across geographies when dressing percentage and the “dressing difference” (visceral fat, blood, and other parts that are generally not consumed by humans) can vary in both quantity and definition by breed and country (30, 31).

Secondly, the FAO collects and disseminates agricultural data from over 245 countries and territories, which includes estimated livestock populations and commodities production approximations (32). These data are compiled by FAOSTAT using reports provided by country governments. The FAOSTAT and DAD-IS databases were selected for use in this project because they harbor a vast amount of data presented in a standardized fashion. However, it is acknowledged that the sources of data vary in collection methodology depending on country of origin, and that where data is missing, FAOSTAT in particular applies extrapolation methods to fill gaps. This may also explain why a comparison of cattle population estimates from DAD-IS with FAOSTAT are not generally found to be identical. Indeed, only six sub-Saharan African countries had >90% identical population estimates when DAD-IS and FAOSTAT data were compared. This may be explained by a small percent of the cattle population in each country falling outside defined breed standards as reported by DAD-IS, but the authors were unable to find a published explanation for this discrepancy. A further limitation related to FAO database estimates surrounds quality of data reporting on carcass weights which are unlikely to be homogenous even within the example countries. It should be highlighted for instance that in our dataset, Niger has an abnormally high dressed carcass weight for 2018. This data was accessed by us in mid-February 2020 and found to be marked as “calculated data.” Therefore, it may change in the database and thus nullify any calculations drawn from it if an error in the data is identified by the publisher.

In Method 3, we attempted to increase granularity by including breed specific data and average bodyweights for populations, combined with assumptions about population structure. In this example, a hypothetical herd structure was applied. The classifying of production systems in sub-Saharan Africa is a large task in itself, and herd structures comprise just one aspect of that (33). To the best of the authors' knowledge, no publicly available repository of data on herd structures within these regions is available at present to support the level of detail proposed here. Therefore, while the authors took advantage of DAD-IS breed data where possible (e.g., available weight data by sex), it was necessary to make use of some herd structure generalizations. This is a limitation of Method 3 which the authors fully acknowledge and propose could be explored in more depth with a sensitivity analysis if more comprehensive data were available. At present, however, given the acknowledged data gaps and a lack of confidence intervals around most of the data published by the FAO, a sensitivity analysis would yield no additional value. Further, the authors note that while the choice to use FAOSTAT and DAD-IS data was made in order to demonstrate facility of the methodologies using widely accepted data repositories, the methodologies discussed in this paper should be considered a demonstration of what could be possible given greater data confidence, rather than a concrete representation of current cattle biomass in the example countries. Thus, estimations of biomass should be re-assessed and may be adapted within these methods as more accurate and detailed population weight estimates and herd structures are made available. Nevertheless, it is believed a few additional corroborating variables introduced as a more structured national herd data collection protocol formulated and disseminated by the FAO could allow aspects of the sector, such as slaughter data, to increase the accuracy of biomass estimation.

Finally, it should be noted that while this paper explores cattle as the model population, the methodologies explored could be similarly applied to other livestock species including small ruminants, pigs, camels, equines, and poultry.

The impetus for considering livestock biomass in the early 20th century was to develop stocking rate estimates for rangeland systems in order to issue recommendations on how many animals could be sustainably grazed on a given area of land. The practice has since evolved in application to underpin critical indicators for food security, public health, and both local and regional economies. As humans and other terrestrial animals, both livestock, and wildlife, come into more frequent and closer contact with each other by nature of finite global landmass and a shared need for adequate nutrition, it has become ever more important that an understanding of physical biomass in a given space can be accurately measured. Given the analysis generated above, it is therefore appropriate to question whether studies that utilize the traditional TLU biomass estimates to support research should be called into question. A potential underestimation of the scale we have demonstrated casts doubt, for example, on whether GHG emissions estimates based on TLUs are accurate, or if measures of feed required to sustain a given population are sufficient. If biomass is miscalculated to the order of magnitude our analysis suggests, the bedrock on which many understandings, policies, and initiatives are built could be questioned. A more precise TLU could substantially enhance food security through more informed livestock production, enhance disease prevention and response capacity, and better equip decision makers in intelligent management of vital ecosystems for equity, sustainability, and biodiversity.

## Data Availability Statement

Publicly available datasets were analyzed in this study. This data can be found here: FAOSTAT: http://www.fao.org/faostat/en/#data and DAD-IS: http://www.fao.org/dad-is/en/.

## Author Contributions

PR-O, JR, and WG conceived the study. PR-O was responsible for drafting the manuscript, designing the methodology, and undertaking the data analysis. JR oversaw the study design and analysis. WG contributed to methodology design and data analysis. All authors contributed to the article and approved the submitted version.

## Conflict of Interest

The authors declare that the research was conducted in the absence of any commercial or financial relationships that could be construed as a potential conflict of interest.
